# Geospatial analysis of spatial distribution, patterns, and relationships of health status in the belt and road initiative

**DOI:** 10.1038/s41598-023-50663-7

**Published:** 2024-01-02

**Authors:** Jie Li, Zejia Xu, Hongxi Wang, Lingling Li, Hong Zhu

**Affiliations:** 1 School of Geographical Sciences and Remote Sensing, Guangzhou University, Guangzhou, 510006 China; 2 Key Laboratory of Philosophy and Social Sciences in Guangdong Province of Maritime Silk Road of Guangzhou University (GD22TWCXGC15), Guangzhou, 510006 China; 3 Guangdong Federation of Social Sciences, Guangzhou, 510000 China

**Keywords:** Health policy, Health services, Public health, Quality of life, Risk factors

## Abstract

The Health Silk Road plays a crucial role in the Belt and Road Initiative, and comprehending the health status within the participating countries is fundamental for fostering cooperation in public health. This paper collected five health indicators to represent the health status of the Belt and Road countries. Employing spatial statistics, the spatial patterns of health indicators and the associations with influencing factors were investigated. The utilized spatial statistics encompass spatial autocorrelation methods, geographical detector and spatial lag model. The results revealed obvious disparities and significant positive spatial autocorrelation of health indicators within the Belt and Road countries. Specifically, countries in Sub-Saharan Africa exhibited significant clustering of limited health indicators, while countries in Europe and Central Asia demonstrated significant clustering of robust health indicators. Furthermore, the health indicators exhibited significant spatial heterogeneity and association with the influencing factors. Universal health coverage, household air pollution, and the prevalence of undernourishment emerge as influential factors affecting health indicators. Overall, our findings highlighted complex influencing factors that contributed to the profound health inequalities across the Belt and Road countries. These factors should be duly considered in public health collaborations within the Belt and Road Initiative.

## Introduction

The Belt and Road Initiative (BRI), encompassing the Silk Road Economic Belt and the 21st Century Maritime Silk Road, is a model of international and regional economic cooperation proposed by China in 2013 to foster deeper development of economic globalization. As of January 2023, 151 countries have signed cooperation documents, highlighting the comprehensive nature of the initiative, which encompasses infrastructure development, economic cooperation and policy dialogue, scientific research, and public health collaboration^1,2^. While economic development is a primary focus, public health is also recognized as a vital aspect of Belt and Road cooperation. The well-being of people and their health are foundational to social and economic development, making cooperation in public health an indispensable part of the initiative.

Since the concept of the "Health Silk Road" was put forward in 2016, China and the participating countries have put great efforts to actively and continuously promote the health of the people of relevant countries. The Health Silk Road aims to provide participating countries with an equal and fair platform for health cooperation and make positive contributions to safeguarding human health. This includes jointly addressing public health challenges, continuously enhancing capabilities in health development, and cooperation related to traditional Chinese medicine^3,4^. In times of public health emergencies, the Health Silk Road establishes rapid response and assistance mechanisms, offering essential technical support and material aid to affected countries. For example, in 2020, China provided epidemic prevention supplies to 150 countries and 13 international organizations and dispatched 37 medical expert teams to 34 countries to combat the COVID-19 pandemic^5^. Meanwhile, the Health Silk Road is committed to support participating countries in bolstering public health infrastructure, to improve the accessibility and quality of public health services^2^. Moreover, China also takes the lead in enhancing the professional capabilities of healthcare personnel in participating countries through medical training, academic exchanges, and technology transfer.

To improve the health status among the participating countries there is still several challenges, including disparities in economic development and health status, the transmission of infectious diseases, and varying local geographic environments and living conditions. The BRI initiative covers 152 countries with a population exceeding 5.2 billion^6^, and there is substantial variation in the health status of people within BRI countries due to disparities in environments and economic development. For instance, the maximum maternal mortality rate within BRI countries exceeds 200 times the minimum^7^. Meanwhile, increased international cooperation has heightened the risk of infectious disease transmission^8^. Infectious diseases can emerge in any BRI country and spread rapidly to otherthrough growing commercial trade and frequent personal exchanges, placing an increasing burden on local public health systems. Furthermore, as most BRI countries are developing countries and heavily reliant on resources for economic growth, severe air and water pollution pose ongoing environmental threats^9^. These diverse health challenges contribute to the complexity and difficulty of public health cooperation in BRI countries.

Addressing these complex challenges necessitates the current conditions of health status in participating countries of BRI, spatial distributions and patterns of different health conditions, and an understanding of the relationships between particular spatial patterns and potential influencing factors to facilitate policy-making. Previous studies have discussed ways of enhancing health cooperation among BRI countries through analyzing the overall health status^4,5,10,11^. However, it is essential to recognize that BRI countries span multiple regions, covering over three-quarters of global countries^1^. Such broad distribution indicates distinct health status and risk factors across regions and countries, warranting a geographical perspective and methodology to explore how local factors influence health status in countries with diverse social and natural conditions.

This study adopts a geographical methodology to analyze five health status indicators and address these public health challenges. There are three central aims of this study. Firstly, it aims to explore the spatial patterns of health status distribution in BRI countries, and compare regional differences through spatial autocorrelation analysis. Secondly, it seeks to reveal the spatial heterogeneity in health status and associations with socioeconomic and environmental factors using geographical detector. Finally, it quantified the effects of socioeconomic and environmental factors on health status using spatial lag model. These aims contribute to showcasing the health status of BRI countries, highlighting public health issues in each country, and thereby facilitating more effective collaboration in the field of health.

## Methods

### Study setting

**Figure 1:**
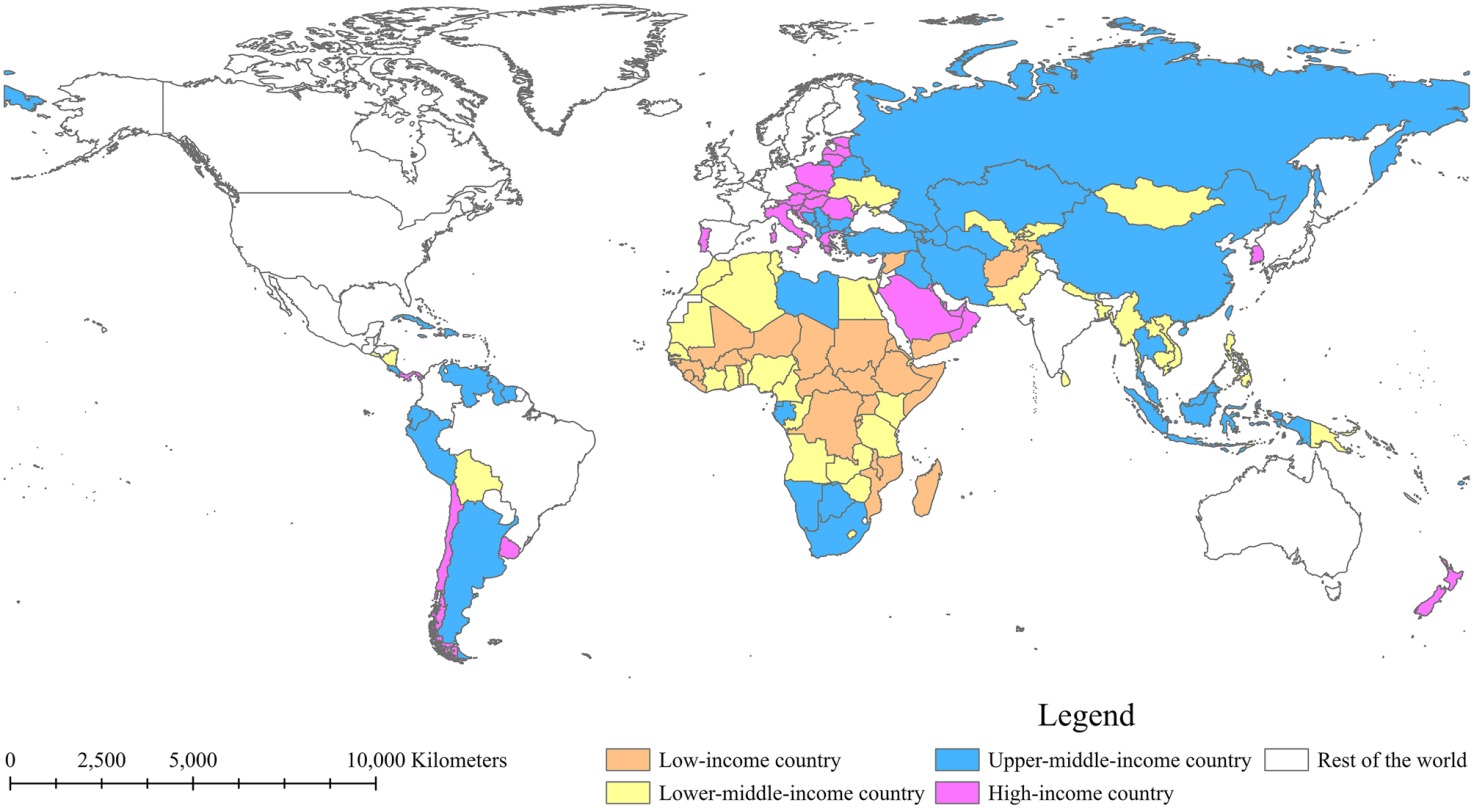
Geographic distribution and income level of the Belt and Road countries in 2023. This map was created by using ArcMap Pro software (https://www.esri.com/en-us/arcgis/products/arcgis-pro/), version 2.8.

**Figure 2:**
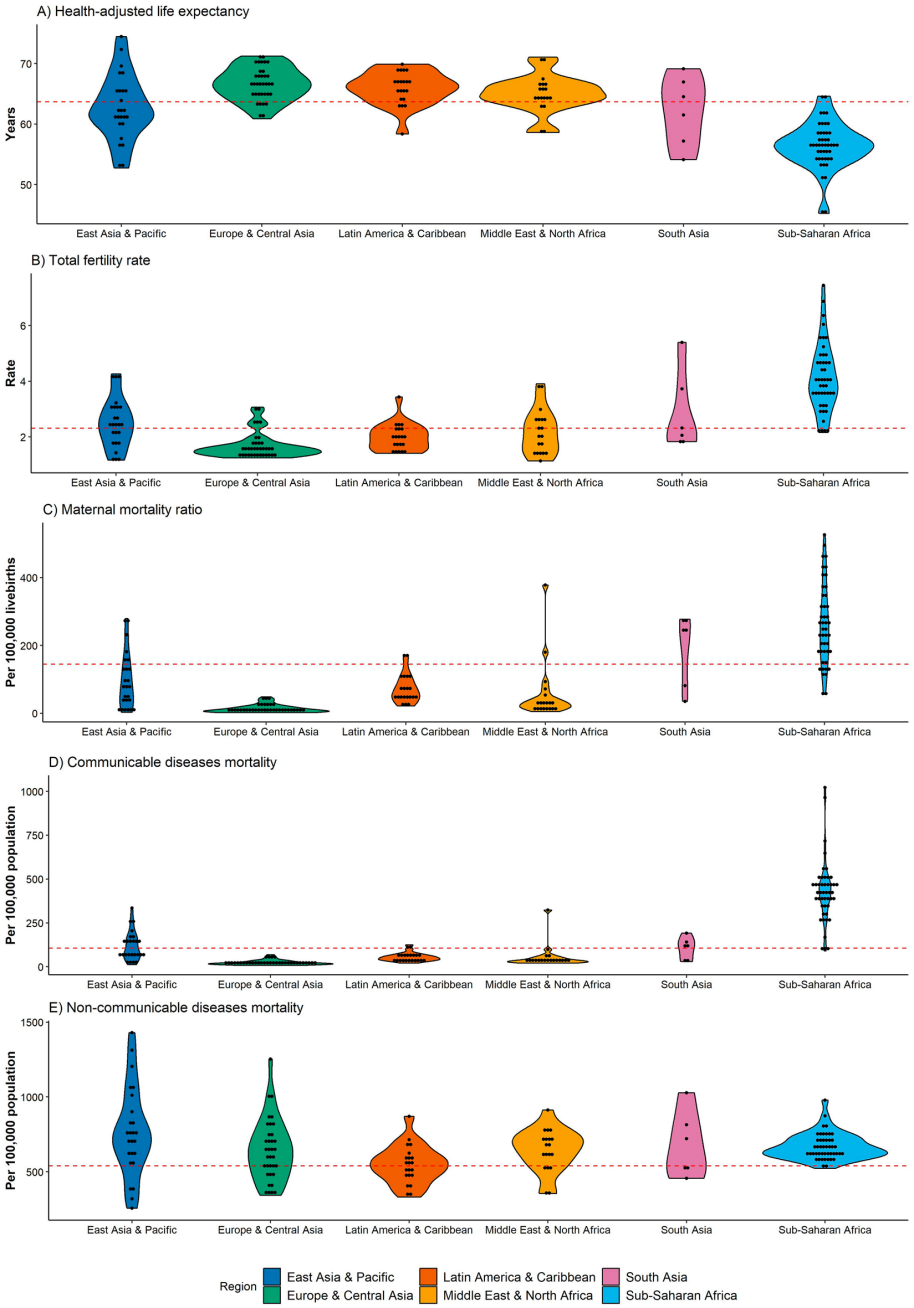
Violin plots of health status in the Belt and Road countries in 2019, by World Bank region (The red dashed line represents the global value for each health status, and spot represents country).

By January 2023, 152 countries worldwide have joined the Belt and Road Initiative, and all countries were selected for analysis in this study^1^. The Belt and Road cooperation includes 52 countries in Africa, 40 countries in Asia, 27 countries in Europe, 11 countries in Oceania, 9 countries in South America, and 12 countries in North America. The majority of the Belt and Road countries are mainly located in Asia and Africa, covering three-quarters of the world’s countries. Meanwhile, to present the socioeconomic status of the BRI countries, countries were divided into 4 income level groups according to the World Bank Atlas method^12^, including low-income countries (LICs), lower-middle-income countries (LMICs), upper-middle-income countries (UMICs) and high-income countries (HICs). The BRI has reached 73 of 79 LICs and LMICs and 79 of 124 UMICs and HICs. The geographic distribution and income level of the BRI countries are shown in Fig. 1. In addition, to illustrate the differences in health status between regions, the 152 BRI countries are categorized into 7 regions according to the World Bank classification^12^ (Fig. 2).

### Health status and indicators

Health status is a composite result that reflects the quality of health services at the country-level. Current public health cooperation under the Belt and Road Initiative faces several challenges, including disparities in health conditions, rising risks of infectious diseases, and diverse health conditions and lifestyles^3,4^. To address these public health challenges, five core indicators in health status were selected, including health-adjusted life expectancy (HALE), total fertility rate (TFR), maternal mortality ratio (MMR), communicable diseases (CD) mortality and non-communicable diseases (NCD) mortality (Table 1). These indicators involve the majority of health status. HALE is the average number of years that a person can expect to live in good health, which can be used to assess the performance of a country’s health systems^13^. The total fertility rate is the mean number of children a woman would have by age 50, which can reflect nutrition, quality and safety of healthcare, and access to health services in a country^14^. Maternal mortality ratio is one of the most important indicators for assessing maternal health and the quality of a country’s health system^15^. Communicable disease mortality is an indicator to assess the control of communicable diseases, which are the leading cause of death and limit life expectancy worldwide^16^. Non-communicable disease mortality is the death rate due to non-communicable diseases such as cardiovascular diseases, cancer, and diabetes, which cause major problems and impose a heavy burden on health in both developed and developing countries^16^.

**Table 1:** Descriptions of health status and health indicators.

Health status	Health indicator	Unit
Life expectancy	Health-adjusted life expectancy	Year
Fertility	Total fertility rate	–
Mortality by cause	Maternal mortality ratio	Per 100,000 live births
	Communicable diseases mortality	Per 100,000 population
	Non-communicable diseases mortality	Per 100,000 population

The health status data was collected from the Global Health Data Exchange database of the Institute for Health Metrics and Evaluation^7^. The health status data in this study include HALE, TFR, MMR, CD mortality, and NCD mortality for the 152 BRI countries in 2019.

### Explanatory variables for health status

The Belt and Road Initiative focuses on promoting socioeconomic cooperation and mutual development among the BRI members. Given this, we focus on the impact of socioeconomic and environmental factors on health status in this study. Based on the indicator framework of the Sustainable Development Goals (SDGs), we selected 6 explanatory variables, referring to basic economic status, access and quality of health system, and people living environment. SDGs are initiatives adopted by the United Nations aimed at addressing global social, economic, and environmental issues. The BRI, as a significant measure by China to promote regional cooperation and development, is closely associated with the United Nations Sustainable Development Goals. Therefore, this study considers SDG indicators as explanatory variables to investigate the potential impact at the country level of achieving SDGs within the BRI framework on the health status of BRI countries.

Data on explanatory variables were collected from multiple sources. Explanatory variables, including per capita government health expenditure (GHE), universal health coverage, prevalence of undernourishment, basic drinking water, and household air pollution were the Global Health Observatory, health dataset of World Health Organization^17^. Urban population ratio was obtained from United Nations World Urbanization Prospects^18^. The list and interpretations of the six factors are shown in Table 2.

**Table 2:** Summary of the six explanatory variables for health status.

Explanatory variable	Interpretation	Unit	SDGs
Per capita GHE	The quality of a country’s health system	1,000 US$	SDG 1.a.2
Universal health coverage	The accessibility of a country’s health system	%	SDG 3.8.1
Prevalence of undernourishment	Proportion of the population living with nutritional deficiencies	%	SDG 2.1.1
Basic drinking water	Population using at least basic drinking water	%	SDG 6.1.1
Household air pollution	Proportion of the population with primary reliance on polluting fuels and technologies for cooking	%	SDG 7.1.2
Urban population ratio	Proportion of urban population to total population	%	–

### Spatial distribution and autocorrelation analysis

Standard deviation ellipse (SDE) is a spatial statistical method for measuring the concentration of spatial features^19^. The main parameters of the SDE include the ellipse center, orientation, long axis, and short axis. We used the SDE to explore the concentration of each health status and compared the ellipses for each health status to reveal the differences in health status. The equation used for the calculation is as follows:

1 $${SDE}_{x}=\sqrt{\frac{\sum_{i=1}^{{\text{n}}}{({{\text{x}}}_{i}-\overline{{\text{x}}})}^{2}}{n}}$$

2 $${SDE}_{y}=\sqrt{\frac{\sum_{i=1}^{{\text{n}}}{({{\text{y}}}_{i}-\overline{{\text{y}}})}^{2}}{n}}$$

where *n* is the total number of countries; ${{\text{x}}}_{i}$ and ${{\text{y}}}_{i}$ are the geographic coordinates of country *i*; $\overline{{\text{x}}}$ and $\overline{{\text{y}}}$ is the average center of countries.

To explore the spatial distribution patterns of health status, spatial autocorrelation analysis was employed. In this study, Global Moran’s *I* and Getis-Ord *G*_i_^*^ were used to reveal the clustering patterns, as well as the distribution of hot spots and cold spots of health status, respectively.

Moran’s *I* is a frequently used spatial statistic for global spatial autocorrelation of geographical features. It characterizes the spatial patterns of spatial features by measuring feature locations and attribute values^20^. We used Moran’s *I* to determine the spatial patterns of health status, whether it is spatial clustering or dispersion. The equation used for the calculation is as follows:

3 $$I=\frac{n}{{\sum }_{i=1}^{n}{\sum }_{j=1}^{n}{W}_{ij}}\frac{{\sum }_{i=1}^{n}\left({x}_{i}-\overline{x }\right){\sum }_{j=1}^{n}{W}_{ij}\left({x}_{j}-\overline{x }\right)}{{\sum }_{i=1}^{n}{\left({x}_{i}-\overline{x }\right)}^{2}}$$

where *n* is the total number of countries; ${x}_{i}$ and ${x}_{j}$ are health status of country *i* and *j* (where *i* ≠ *j*); $\overline{{\text{x}}}$ is the average over all locations of countries; ${W}_{ij}$ is the spatial weight between countries *i* and *j*. The value of Moran’s *I* lie between [− 1, 1]; when Moran’s *I* > 0, the health status is spatial clustering; when Moran’s *I* < 0, the health status is spatial dispersion; when Moran’s *I* = 0, the health status is randomly distributed. The *Z*-score and *P*-value provide statistical significance on the calculated Moran’s *I* using a 95% confidence level.

Getis-Ord *G*_i_^*^ is a spatial statistic that analyzes the local spatial pattern of a feature. The *G*_i_^*^ statistic, on the other hand, is the local spatial autocorrelation index. It determines whether the clustering pattern of features is high- or low-value concentration^21^. The equation used for the calculation is as follows:

4 $${G}_{i}^{*}= \frac{{\sum }_{j=1}^{n}{W}_{ij}{x}_{j}-\overline{x}{\sum  }_{j=1}^{n}{W}_{ij}}{{\sum }_{j}^{n}{W}_{ij}{x}_{j}}$$

where ${W}_{ij}$ is the spatial weight between countries *i* and *j*. Positive *G*_i_^*^ indicates that country *i* is surrounded by countries with high values of health status, and the country is regarded as a hot spot; negative *G*_i_^*^ indicates that country *i* is surrounded by countries with low values of health status, and the country is regarded as a cold spot.

Statistical analyses were performed using the R (v4.3.0, https://www.r-project.org/). Spatial analyses, including, SDE, Global Moran’s *I*, and Getis-Ord G_i_^*^, were implemented in ArcGIS Pro (v2.8, ESRI, Redlands, CA, USA).

### Spatial correlation analysis

To quantify the spatial stratified heterogeneity and assess the spatial correlation for explanatory variables of health status in the BRI countries, we used geographical detector. This statistical tool is designed for detecting spatial stratified heterogeneity (SSH) within spatial features^22^. It evaluates whether a spatial feature contributes significantly to the observed spatial patterns of health indicators. Unlike methods requiring linear hypotheses, the geographical detector does not rely on such assumptions. This assessment is calculated from the difference between the dispersion variances among stratified populations. The equation for the calculation is as follows:

5 $$q=1-\frac{\sum_{h=1}^{L}{N}_{h}{\sigma }_{h}^{2}}{N{\sigma }^{2}}$$

where *N* is the number of units in the study area; ${\sigma }^{2}$ is the variance of factor in the study area; *h* = 1, 2, …, *L* is strata of variable. The value of the *q*-statistic lies between [0, 1]; when *q*-statistic = 0, the explanatory power of a given factor is not significant; when *q*-statistic = 1, the explanatory power of a given factor is perfect. To perform the analysis in the geographical detector, we reclassified factors into 5 levels using the natural break classification method. Spatial correlation analysis was conducted utilizing Geodetector software (http://geodetector.cn/).

### Spatial regression analysis

To explore the effect of socioeconomic and environmental factors on health status, spatial lag model (SLM) was used to reveal the associations. In the spatial lag model, all independent variables are included, rather than being limited to a single independent variable. This allows the model to consider the spatial interdependence of the dependent variable, meaning that the dependent variable is influenced by the values of independent variables in neighboring geographical units. Spatial lag model is a spatial regression model applied to quantify the effect of long-term stable factors, such as socioeconomic status, local geographic environment, and living condition^23,24^. The equation for the calculation is as follows:

6 $${s}_{i}=\rho {W}_{{s}_{i}}+X\beta +\varnothing $$

where ${s}_{i}$ is the dependent variable for a specific location. $\rho$ is the spatial autoregressive coefficient of the lag term, which measures the extent to which the value of ${s}_{i}$ in a location is influenced by the values of ${s}_{i}$ in neighboring locations. $W$ is the spatial adjacent matrix, reflecting the spatial trend of the response variables. $X$ are all selected explanatory variables in this study. $\beta$ is the spatial regression coefficient of the explanatory variables. $\varnothing$ is the error term of the spatial autocorrelation. Spatial lag model was performed using GeoDa software (v1.20, http://geodacenter.github.io/).

## Results

### Spatial variation characteristics

The original values and spatial variation of health status indicators in BRI countries in 2019 were depicted in Table S1 and Fig. 2, respectively. As of January 2023, the BRI has expanded to cover over three-quarters of countries worldwide. Within the BRI countries, there is significant variation in socioeconomic status. Similarly, health status indicators also exhibit distinct variation characteristics across BRI countries in 2019.

The distribution characteristics of HALE show similarities across regions (Fig. 2). While more than half of BRI countries have higher HALE than the global average, the relatively low HALE in East Asia and Pacific and Sub-Saharan Africa is noteworthy. Over half of HALE in East Asia and Pacific and almost all of HALE in Sub-Saharan Africa were below the global HALE. In the remaining regions, HALE in most countries exceeded the global average. The relatively high HALE was predominantly observed in Europe and Latin America. At the country level, HALE ranged from 45.24 years in Lesotho to 74.48 years in Singapore, representing a difference of almost 1.6 times (Table S1).

TFR in BRI countries display exactly opposite variation characteristics compared to HALE. Over half of TFR in East Asia and Pacific and almost all of TFR in Sub-Saharan Africa were above the global average, while most of the remaining regions were below the global average. Notably, most of TFRs in Europe and Central Asia were concentrated at significantly lower levels, whereas TFRs in other regions were dispersed. At the country level, TFR varied widely across BRI countries, ranging from 1.14 in the United Arab Emirates to 7.44 in Niger, corresponding to a variation of more than 6 times.

The distribution of MMR demonstrates significant variation between Sub-Saharan Africa and other regions. In general, MMRs in 101 of 152 BRI countries were below the global average, with the majority of these countries located in regions other than Sub-Saharan Africa. Notably, all MMR in Europe and Central Asia was concentrated at significantly lower levels, with a regional average of less than 10% of the global average. In contrast, most of the relatively high MMRs were observed in Sub-Saharan Africa. The average MMR in Sub-Saharan Africa was more than 1.7 times the global average and 15 times the average in Europe and Central Asia. At the country level, MMR ranged from 2.07 per 100,000 livebirths in Cyprus to 526.21 per 100,000 livebirths in Liberia, corresponding to a variation of more than 250 times.

CD mortality in BRI countries displays a significantly concentrated distribution characteristic. In general, CD mortality was less than the global average in Europe and Central Asia, Latin America and Caribbean, and Middle East and North Africa, while high CD mortality was concentrated in Sub-Saharan Africa. Notably, CD mortality in Europe and Central Asia was extremely low, with a regional average of 20.40 per 100,000 population. In contrast, almost all CD mortality rates in Sub-Saharan Africa were more than the global average, with a regional average of 426.11 per 100,000 population. This was more than 200 times the average for Europe and Central Asia. At the country level, CD mortality rates varied from 7.66 per 100,000 population in Austria to 1022.40 per 100,000 population in Lesotho, corresponding to a variation of more than 130 times.

Compared to CD mortality, NCD mortality exhibited similar distribution characteristics across regions. In 119 of 152 BRI countries, NCD mortality was higher than the global average, with relatively high NCD mortality rates observed in all regions. Notably, although all NCD mortality in Sub-Saharan Africa exceeded the global average, the average mortality in Sub-Saharan Africa was only slightly higher than that in Europe and Central Asia. NCD mortality rates in East Asia and Pacific were relatively high among BRI countries. Additionally, NCD mortality rates in BRI countries varied from 256.00 per 100,000 population in Singapore to 1429.08 per 100,000 population in the Solomon Islands, both distributed in East Asia and Pacific.

**Figure 3:**
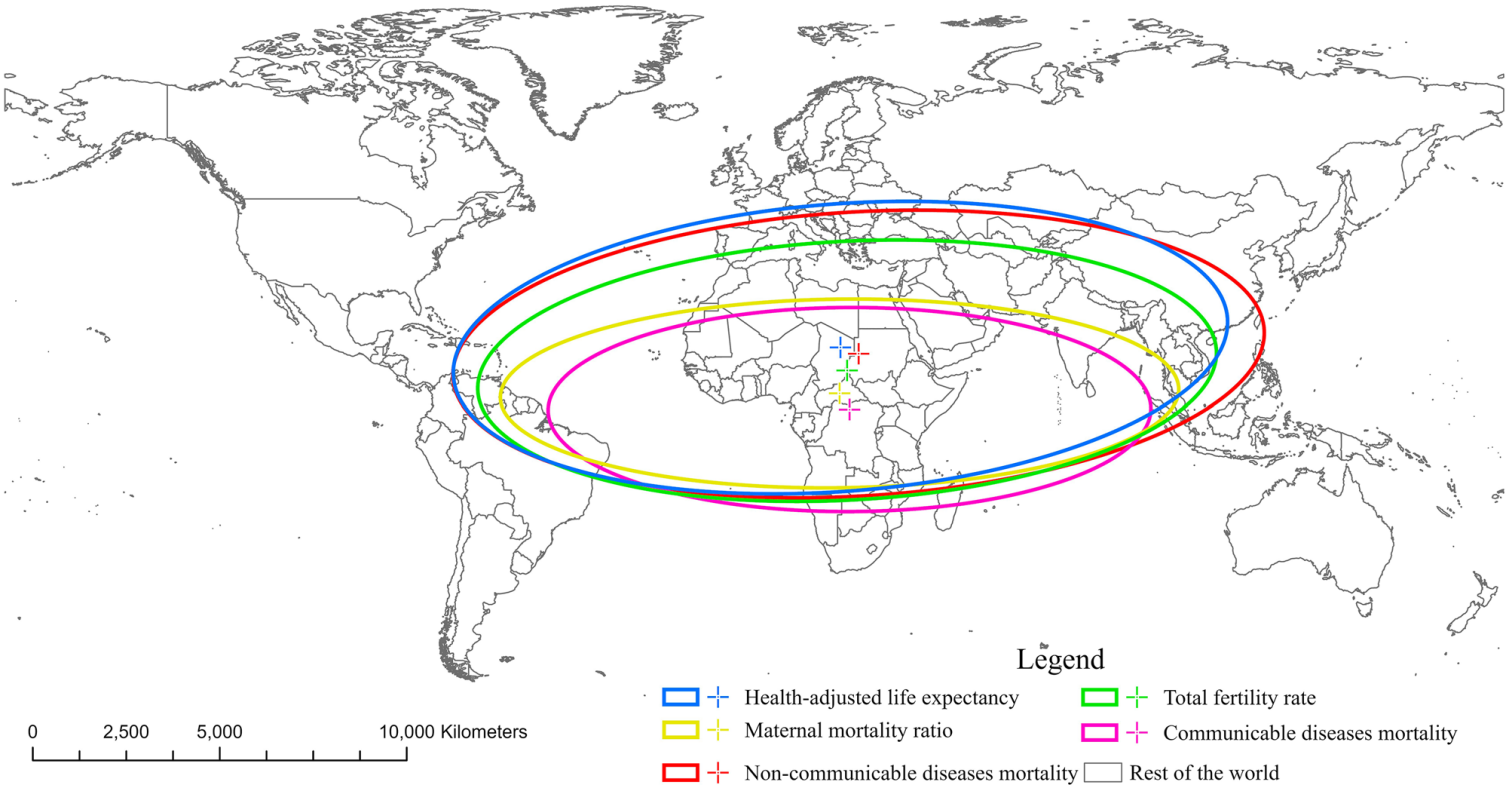
Standard deviation ellipse analysis of health status in the Belt and Road countries in 2019. This map was created by using ArcMap Pro software (https://www.esri.com/en-us/arcgis/products/arcgis-pro/), version 2.8.

Furthermore, this study utilizes standard deviation ellipses to elucidate the spatial variation of health status indicators among BRI countries in 2019 (Fig. 3). The standard deviation ellipse provides valuable insights into the concentration patterns of health status, leading to several key observations. Notably, the ellipse of HALE encompasses a wide range of BRI countries, with the ellipse center positioned furthest north in comparison to other ellipses. This finding indicates a relatively slight variation in HALE among BRI countries, with a broad prevalence of high HALE values across the countries. Likewise, a similar trend emerges in the ellipse for NCD mortality. The ellipse for NCD mortality exhibits an even broader coverage, particularly in the eastern direction, signifying the widespread prevalence of elevated NCD mortality rates, notably in East Asia and Pacific. Comparatively, the ellipse for TFR exhibits a narrower coverage compared to HALE and NCD mortality, with the ellipse center further south. This finding reveals a broad distribution of relatively high TFRs within BRI countries, particularly in regions covered by the ellipse, such as Sub-Saharan Africa, where significantly higher TFRs are observed. In addition, the ellipses for MMR and CD mortality exhibit similar concentration patterns. The ellipses are significantly concentrated in Sub-Saharan Africa, with the ellipse center positioned furthest south. This finding indicates a significant concentration of high MMR and CD mortality rates in Sub-Saharan Africa, with notable variations compared to other regions.

### Spatial autocorrelation characteristics

The global spatial autocorrelation and significance levels for the five health indicators of the BRI countries are presented in Table 3. The Moran’s *I* for all five health indicators were all positive and statistically significant at the *P* < 0.01 level, indicating significant spatial clustering of these health indicators within BRI countries. Countries tend to be neighboring countries with similar values of health indicators. Furthermore, Moran’s *I* values for the five health indicators revealed the following clustering patterns: CD mortality > HALE > MMR > TFR > NCD mortality. This finding suggests that HALE, MMR, and TFR exhibit similar levels of spatial clustering, with CD mortality rates more likely to exhibit considerable clustering, while the clustering of NCD mortality was relatively weaker.

**Table 3:** Global Moran’s *I* of health status of the Belt and Road countries in 2019.

Health status indicators	Moran's *I*	*Z*-score	*P*-value	Spatial pattern
Health-adjusted life expectancy	0.486	18.607	< 0.001	Clustering
Total fertility rate	0.421	16.204	< 0.001	Clustering
Maternal mortality ratio	0.433	16.645	< 0.001	Clustering
Communicable diseases mortality	0.574	22.148	< 0.001	Clustering
Non-communicable diseases mortality	0.261	10.229	< 0.001	Clustering

**Figure 4:**
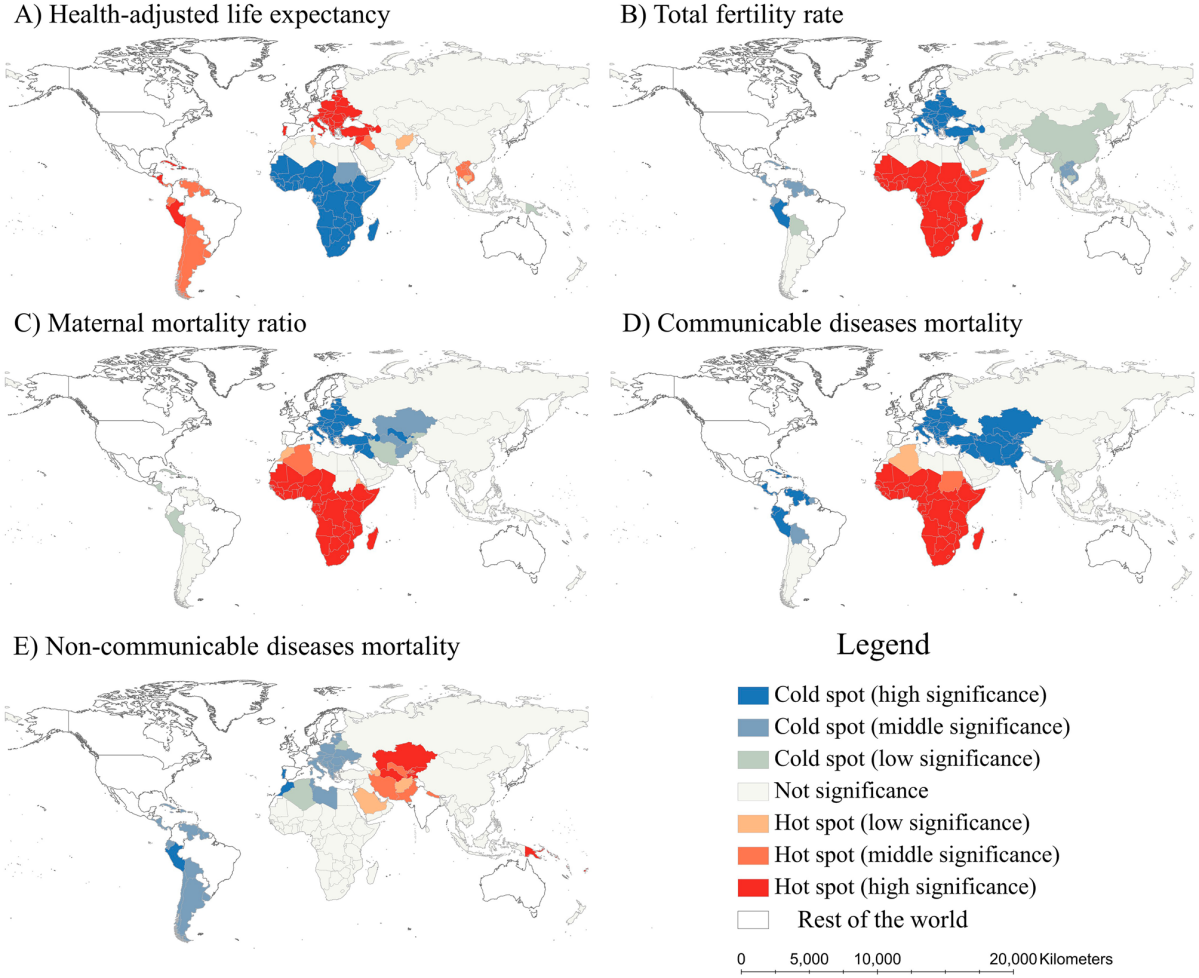
Hot and cold spots of health status in the Belt and Road countries in 2019. This map was created by using ArcMap Pro software (https://www.esri.com/en-us/arcgis/products/arcgis-pro/), version 2.8.

This study employs the Getis-Ord *G*_i_^*^ to examine the local spatial autocorrelation of five health indicators, represented by hot and cold spots (Fig. 4). The cold spots (low values clustering) for HALE were primarily observed in Sub-Saharan Africa, while the hot spots (high values clustering) were identified in Europe and Central Asia and Latin America and Caribbean with high statistical significance. Conversely, the clustering pattern for TFR was precisely the opposite of that for HALE. The hot spots for TFR were predominantly concentrated in Sub-Saharan Africa, while the cold spots were clustered in Europe and Central Asia, Latin America and Caribbean, and East Asia. Furthermore, MMR and CD mortality exhibited similar spatial clustering patterns. The hot spots for these indicators were concentrated in all countries within Sub-Saharan Africa, while cold spots were concentrated in all countries within Europe and Central Asia. Notably, the hot and cold spots for NCD mortality were relatively dispersed and exhibited lower significance levels compared to other health indicators. The hot spots were primarily concentrated in Central Asia, Western Asia, and Pacific islands, while the cold spots were distributed across Europe, North Africa, and Latin America and Caribbean.

### Spatial correlation analysis

The results of the spatial heterogeneity analysis for the five health indicators of the BRI countries are provided in Table 4. The majority of *Q*-statistic values for the health indicators demonstrated significant correlations (*P* < 0.01) with socioeconomic and environmental factors, indicating substantial relationships. Generally, except for NCD mortality, the *Q*-statistic values ranged from 0.3 to 0.7, indicating a noteworthy degree of explanatory power. Among the socioeconomic and environmental factors, universal health coverage exerted the greatest influence, closely followed by household air pollution, while the prevalence of undernourishment exhibited the lowest impact. Meanwhile, the *Q*-statistic values for per capita GHE, basic drinking water, and urban population ratio varied slightly across the health indicators, yet still exhibited relatively considerable correlations. Notably, the association of socioeconomic and environmental factors with NCD mortality differed from other health indicators. The *Q*-statistic values for factors associated with NCD mortality ranged from 0.198 to 0.349, indicating a lower magnitude compared to the values associated with other health indicators. The effect of the prevalence of undernourishment on NCD mortality was not statistically significant at the *P* > 0.05 level. These findings indicated that the mechanisms influencing NCD mortality are more multifaceted and intricate in comparison to other health indicators.

**Table 4:** The *Q*-statistic of the 6 explanatory variables for health status by geographical detector.

Explanatory variable	*Q*-statistic				
	Health-adjusted life expectancy	Total fertility rate	Maternal mortality ratio	Communicable disease mortality	Non-communicable disease mortality
Per capita GHE	0.527**	0.499**	0.445**	0.392**	0.327**
Universal health coverage	0.692**	0.702**	0.697**	0.683**	0.349**
Prevalence of undernourishment	0.319**	0.303**	0.364**	0.286**	0.081
Basic drinking water	0.559**	0.585**	0.502**	0.553**	0.240**
Household air pollution	0.631**	0.629**	0.696**	0.683**	0.198*
Urban population ratio	0.579**	0.553**	0.496**	0.522**	0.201**

**Statistical significance level: 0.01.

*Statistical significance level: 0.05.

### Spatial regression analysis

Table 5 presents the regression coefficients and significance levels of socioeconomic and environmental factors for the health indicators of the BRI countries. The results of HALE indicate that per capita GHE and universal health coverage presented a positive and significant influence on HALE, while the prevalence of undernourishment and household air pollution demonstrated a negative and significant influence. Specifically, a 1% increase in universal health coverage corresponds to a rise of 0.121 years in HALE, whereas a 1% increase in prevalence of undernourishment relates to a decrease of 0.068 years in HALE.

**Table 5:** Regression coefficients of the 6 explanatory variables for health status using spatial lag model.

Explanatory variable	Coefficient				
	Health-adjusted life expectancy	Total fertility rate	Maternal mortality ratio	Communicable disease mortality	Non-communicable disease mortality
Per capita GHE	0.881**	0.020	− 2.650	11.481	− 75.890**
Universal health coverage	0.121**	− 0.037**	− 2.016**	− 2.780**	− 1.879
Prevalence of undernourishment	− 0.068**	0.009	2.091**	3.296**	− 0.307
Basic drinking water	0.016	− 0.005	− 0.468	− 1.249**	0.711
Household air pollution	− 0.046**	0.012**	1.753**	2.683**	− 0.317
Urban population ratio	0.009	− 0.001	0.592	0.802	− 1.605*

**Statistical significance level: 0.01.

*Statistical significance level: 0.05.

Regarding TFR, the results reveal a significant negative influence on universal health coverage and a positive influence on household air pollution. Meanwhile, MMR and CD mortality present similar influencing factors. Universal Health Coverage exhibits a negative influence on both MMR and CD mortality, while the prevalence of undernourishment and household air pollution demonstrate positive influences. In addition, basic drinking water exhibits a negative influence on CD mortality.

Furthermore, per capita GHE and the urban population ratio show a negative influence on NCD mortality. Specifically, a 1000$ increase in per capita GHE is associated with a decrease of 75.890 in NCD mortality, and a 1% increase in urban population ratio corresponds to a decrease of 1.605 in NCD mortality.

## Discussion

Understanding the health status within the Belt and Road Initiative countries serves as a fundamental basis for fostering further collaborations in public health. This study employed spatial statistics to investigate the distribution, autocorrelation, and socioeconomic and environmental factors influencing health status across BRI countries in 2019. The findings reveal substantial disparities in the distribution of health status among the BRI countries. Countries with robust economic conditions, such as those in Europe, South America, and East Asia, exhibit clusters of high HALE. Conversely, countries with poorer economic conditions, particularly in Sub-Saharan Africa, exhibit clusters of high TFR, MMR, and CD mortality. NCD mortality demonstrates a widespread distribution and clustering across the BRI countries, indicating the intricate nature of such diseases. Furthermore, this study elucidated the spatial correlation effects of health indicators, considering the influence of several socioeconomic and environmental factors, including per capita GHE, universal health coverage, and access to basic drinking water.

Spatial disparities in both economic conditions and health status indicators within the BRI countries are significant and complex^25^. As of January 2023, participation in the BRI encompasses three-quarters of countries worldwide, including the poorest countries, developing countries with great potential, and many developed countries. This diversity of economic conditions was also reflected in the health status indicators. The greatest disparity in HALE reached approximately 30 years, while the largest differences in MMR and CD mortality even exceed 250-fold and 130-fold, respectively. Overall, Sub-Saharan Africa, the impoverished region, exhibited poor health status, characterized by the lowest average HALE and the highest MMR and CD mortality. This situation arose from a combination of multifaceted factors, including widespread infectious diseases, undernutrition, inadequate healthcare services, and particularly limited economic development^26,27^. Furthermore, notable health disparities within East Asia and Pacific were also evident, with significant variations observed across all health indicators. The vast social, economic, and political diversity of the region contributes to the divergent nature of the health system and disparate health status^28,29^.

Overall, there are substantial disparities and spatial clustering in health status within BRI countries, including life expectancy, fertility, and mortality by cause. Given these variations, countries engaging in public health cooperation within the BRI should tailor their approaches to the unique health status and governance models, selecting cooperation strategies aligned with their national contexts. Meanwhile, this study demonstrates that notable public health challenges are often regional rather than isolated to individual countries. Countries within the same region should collaborate to address shared public health challenges, while countries outside the region can provide relevant experiences and assistance. Additionally, the results exhibited that Sub-Saharan African countries generally experience lower life expectancy and elevated mortality rates. Intense survival pressure, coupled with inadequate sanitation conditions, contributes to high fertility rates and maternal mortality in these countries. Social inequalities, infections, and the impacts of wars and famines further lead to heightened mortality rates from various diseases in Sub-Saharan African countries. Many countries in this region are grappling with addressing issues of food and nutrition security. Given that economic development is often prioritized to address these concerns, allocating additional funds to the health sector becomes challenging. Therefore, collaborative efforts with these countries should prioritize economic cooperation to meet the basic food and nutrition needs of the population. For countries in tropical climate regions, infectious diseases are still the major cause of diseases. Therefore, public health resources should be more targeted to cure and prevent infectious diseases, by improving universal coverage of vaccines and cleaning the environment for example. For European countries, NCD is more of a concern than infectious diseases. Coupled with the information that European countries generally have higher life expectance, it can be deduced that European countries generally have higher standards of health care, and people mostly die of aging. However, prevention of unknown infectious diseases such as COVID-19 is still one of the priorities for European countries, as for all other countries. As COVID-19 have shown, an unknown infectious disease can spread easily globally within a short period, and calls for collaboration between all participating countries as well as all other countries to prevent future unknown infectious diseases from causing global pandemic. Simultaneously, endeavors should be directed toward building healthcare infrastructure to fulfill fundamental health requirements and elevate universal health coverage.

According to this study, a variety of factors exerted significant influences on HALE changes, including per capita GHE, universal health coverage, prevalence of undernourishment, and household air pollution. Per capita GHE demonstrates a significant influence on life expectancy in both developed and developing countries^30^, and in turn, life expectancy impacts economic growth in developing countries^31^. Universal health coverage plays a crucial role in enhancing HALE and reducing health inequalities in life expectancy^32^. Using spatial lag model, a 1% increase in universal health coverage corresponds to a gain of 0.121 years in HALE. This is a cost-effective approach to increase HALE among socioeconomic factors, particularly in countries where progress in life expectancy has been limited. In reality, the improvement of universal health coverage is often associated with socioeconomic and health service factors, such as per capita GHE in this study. GHE is one of the primary sources for achieving universal health coverage, and governments typically need to allocate more funds to expand the coverage of medical services^33^. The detrimental effects of undernourishment on HALE are significant and multifaceted, including stunted growth, weakened immune system, and increased susceptibility to diseases^34^. These health consequences contributed to higher mortality and diminished life expectancy. Regrettably, undernourishment remains a persistent public health challenge, particularly in low-income countries, with South Asia and Sub-Saharan Africa being disproportionately affected^35^. Furthermore, household air pollution has significant influences on HALE and serves as an important contributor to the reduction in life expectancy^36^. Household air pollution predominantly arises from the use of polluting fuels and technologies for cooking and heating. Although the reliance on polluting fuels has gradually decreased worldwide due to economic growth, many populations in developing countries, particularly in Sub-Saharan Africa, continue to heavily rely on these polluting fuels for daily living^37^.

The influence of socioeconomic and environmental factors on TFR and MMR, two fundamental indicators of population growth, warrants attention. TFR and MMR exhibited significant negative associations with universal health coverage and significant positive associations with household air pollution. In addition, MMR demonstrates a significant negative association with the prevalence of undernourishment. Previous studies have demonstrated that universal health coverage positively influences maternal health and reduces maternal mortality at the individual level, whereas household air pollution and undernourishment have adverse effects on maternal health^38,39^. At the country level, universal health coverage reflects the capacity of national healthcare systems, while household air pollution and prevalence of undernourishment reflect the quality of life for populations^40^. Countries with limited economic conditions, particularly in Sub-Saharan Africa, generally exhibit challenges in these indicators. In these countries, individuals often face immense pressures for survival while experiencing limited productivity, which leads to a necessity for larger household sizes^39,41^. However, these individuals simultaneously encounter multiple risks from environmental pollution and inadequate nutrition. These contributed to the geographic clustering of high TFR and MMR in developing countries, particularly in Sub-Saharan Africa.

Similar to HALE, CD mortality is also influenced by multifaceted socioeconomic and environmental factors. Universal health coverage and access to basic drinking water exhibited significantly positive influences on CD mortality, while household air pollution and undernourishment have adverse effects. Previous studies have illustrated that adequate universal health coverage plays a vital role in protecting the public from the impacts of infectious diseases and averting premature deaths^42,43^. Access to safe drinking water and sanitation services has effectively prevented the transmission of pathogens and viruses that cause infectious diseases^44,45^. Conversely, undernourishment weakens the immune system, making individuals more vulnerable to infections and increasing the risk of severe outcomes^34,46^. Prolonged exposure to household air pollution irritates the respiratory system, weakens the immune response, and heightens susceptibility to infections such as pneumonia, bronchitis, and tuberculosis^47,48^. However, less developed countries cannot generally effectively address the multiple challenges posed by communicable diseases. The combination of these factors ultimately contributed to the enormous spatial disparities and significant clustering of CD mortality within the BRI countries.

Non-communicable diseases also pose a significant burden within the BRI countries. NCDs, such as diabetes, cancer, and cardiovascular disease, are the leading cause of death globally in both developed and developing countries, requiring long-term diagnostic and substantial therapeutic resources^49,50^. Our study showed that NCD mortality rates within the BRI countries were higher than the global average in general. However, there are significant differences in NCD mortality rates between countries^51^. Countries with higher income levels tend to exhibit a higher prevalence of risk factors for NCD. Conversely, countries with lower income levels face limitations in accessing effective prevention and treatment resources for NCDs. These factors collectively contributed to the higher NCD mortality rates observed within the BRI countries. Furthermore, the mechanisms influencing NCD mortality are more intricate in comparison to other health indicators. NCD mortality is negatively influenced by per capita GHE and urban population ratio. Sufficient GHE can enhance the capacity to address NCDs and reduce associated mortality rates, through improving access to healthcare services and providing long-term treatment and management^49,52^. Meanwhile, a higher urban population ratio generally indicates greater urbanization, which further represents higher individual income and a more sufficient healthcare infrastructure^53,54^.

As a result, these findings highlight the several noteworthy spatial relationships between SDGs indicators and basic health status within BRI. Notably, SDG indicators primarily influence health status indicators such as HALE, MMR, and CD mortality. These health outcomes reflect the major public health challenges in many developing countries, especially in Sub-Saharan Africa. Several SDG indicators, those considered in this study, exhibit considerable associations with social and economic development^32,52^, including universal health coverage, air pollution or undernourishment. These indicators often depict challenges prevalent in developing countries. This observation suggests a close connection between economic development and the enhancement of health-related SDG indicators. Meanwhile, economic cooperation emerges as a pivotal aspect of the BRI. Therefore, the BRI should focus on economic cooperation to address sustainable development issues in developing countries, ultimately contributing to the achievement of health-related SDGs and the improvement of fundamental health status.

This study has several limitations that should be acknowledged. Firstly, the spatial statistical analysis conducted in this study focused on exploring spatial autocorrelation and associations at the country level. However, it is important to note that spatial association and heterogeneity also exist within countries, particularly in those with vast geographical areas. Future studies could benefit from conducting spatial analysis at the sub-national levels to gain a deeper understanding of the association between health indicators and contributing factors. Secondly, this study did not include predictive analysis of spatiotemporal trends in health status. Investigating such trends would provide valuable insights into the dynamics and changes in health indicators over time. Furthermore, the availability and quality of data presented challenges in this study, preventing the inclusion of certain health indicators such as the number of physicians, education levels, and housing conditions. To enhance the robustness of future studies, efforts should be made to collect and analyze data encompassing a wider range of indicators. Overall, while this study provides important insights into the spatial analysis of health status, it is essential to recognize and address these limitations to further advance our understanding of the complex relationships between health status and contributing factors. In future research, it is crucial to conduct in-depth investigations into the distribution patterns of individual health indicators across various regions, countries, and within countries, along with their local influencing factors. Additionally, understanding how local public health development can be integrated into health cooperation initiatives under the Belt and Road Initiative is essential.

## Conclusion

This study explored the spatial patterns of health status and the associations with contributing factors within the Belt and Road Initiative countries. Overall, there are considerable spatial disparities and profound health inequalities in health status across the BRI countries. Our results indicate that theses disparities in health status mainly stem from the national variations in social, economic and environmental factors that are strongly correlated with economic growth. Generally, robust and significantly clustered health status indicators are observed in countries with higher income levels, while limited and significantly clustered health indicators are found in countries with lower income levels. Based on these findings, there are several considerations for collaborations in public health in the BRI. Local health status and economic conditions should be taken into account when designing collaborative efforts. Countries with lower income levels should focus on fostering economic growth and developing healthcare infrastructure. Conversely, countries with higher income levels should emphasize the exchange of experiences in public health, particularly in the prevention and treatment of non-communicable diseases.

Furthermore, it is important to recognize that the Belt and Road Initiatives is not solely a cooperation between China and the BRI countries, but also a platform for collaboration among the BRI countries. The BRI should establish an evaluation system for health standards and promote the rapid development of health services across BRI countries. Simultaneously, the BRI should strengthen the development of health platforms and enhance the effectiveness of mutually beneficial cooperation. Currently, there are still no specific schemes on what levels of health participating countries should reach, how these countries should collaborate on specific aspects of health promotion, and timelines for these efforts. This study provides a basis for these efforts, which characterized the spatial pattern of health status in BRI countries and explored their potential influencing factors. The next steps of research could take several directions. First, to analyze spatiotemporal changes in the health status in participating countries and to compare the change; second, to compare the health status of BRI countries with SDGs, and to quantify the differences to facilitate more targeted efforts. Third, to construct a comprehensive scheme to evaluate the health status of participating countries, as well as countries globally. Last but not least, future research could delve deeper into the analysis of health challenges at regional and sub-national levels, and discuss how to integrate regional public health development into the overall development of the Belt and Road Initiative.

### Supplementary Information

Supplementary Tables.

## Data Availability

Data are available in a public, open access repository. See: https://www.who.int/data/gho and https://vizhub.healthdata.org/gbd-results/.
