# Supplementary material for: Geospatial analysis of spatial distribution, patterns, and relationships of health status in the belt and road initiative
Source: Sci Rep. 2024 Jan 2;14:204. doi: 10.1038/s41598-023-50663-7 (PMC10761736; doi:10.1038/s41598-023-50663-7)
Supplement: Supplementary file 1 — Supplementary Tables. [file 41598_2023_50663_MOESM1_ESM.docx]

**Supplemental Materials**

**Supplementary Table 1.** The original values of health status in the Belt and Road countries in 2019, by location.

| **Country** | **Income level** | **Health-adjusted life expectancy (Years)** | **Total fertility rate** | **Maternal mortality ratio (per 100,000 livebirths)** | **Communicable disease mortality (per 100,000 population)** | **Non-communicable disease mortality (per 100,000 population)** |
| --- | --- | --- | --- | --- | --- | --- |
| ***Global*** | | 63.69 | 2.31 | 145.16 | 105.63 | 539.62 |
| *East Asia & Pacific* | | 62.81 | 2.54 | 88.24 | 110.72 | 814.29 |
| Brunei Darussalam | High | 65.26 | 1.71 | 38.30 | 69.97 | 746.06 |
| Cambodia | Lower-middle | 61.20 | 2.59 | 129.29 | 205.25 | 706.88 |
| China | Upper-middle | 68.53 | 1.43 | 10.3 | 20.17 | 562.25 |
| Cook Islands | High | 65.29 | 2.23 | 3.61 | 59.34 | 610.93 |
| Fiji | Upper-middle | 59.66 | 2.53 | 87.12 | 73.96 | 1011.44 |
| Indonesia | Upper-middle | 62.55 | 1.86 | 137.16 | 128.57 | 745.60 |
| Kiribati | Lower-middle | 53.54 | 3.09 | 265.30 | 270.45 | 1312.36 |
| Lao People's Democratic Republic | Lower-middle | 60.71 | 2.76 | 181.31 | 168.65 | 764.88 |
| Malaysia | Upper-middle | 65.65 | 2.08 | 47.29 | 128.41 | 555.25 |
| Micronesia | Lower-middle | 56.68 | 2.53 | 122.10 | 161.98 | 1203.99 |
| Mongolia | Lower-middle | 60.44 | 3.02 | 49.83 | 37.59 | 1066.05 |
| Myanmar | Lower-middle | 60.85 | 2.35 | 156.66 | 132.65 | 774.22 |
| New Zealand | High | 69.59 | 2.08 | 9.77 | 12.04 | 381.88 |
| Niue | High | 61.36 | 2.39 | 71.50 | 69.72 | 827.21 |
| Papua New Guinea | Lower-middle | 56.36 | 4.27 | 231.88 | 245.48 | 901.28 |
| Philippines | Lower-middle | 62.73 | 2.98 | 69.74 | 157.82 | 635.11 |
| Republic of Korea | High | 72.33 | 1.22 | 9.28 | 26.27 | 319.02 |
| Samoa | Upper-middle | 61.82 | 2.38 | 30.72 | 83.45 | 825.12 |
| Singapore | High | 74.48 | 1.16 | 2.87 | 52.88 | 256.00 |
| Solomon Islands | Lower-middle | 52.73 | 4.22 | 279.44 | 334.60 | 1429.08 |
| Thailand | Upper-middle | 68.40 | 1.20 | 48.23 | 74.13 | 389.60 |
| Timor-Leste | Lower-middle | 61.65 | 4.06 | 158.93 | 175.35 | 713.97 |
| Tonga | Upper-middle | 63.91 | 3.17 | 89.03 | 77.19 | 692.94 |
| Vanuatu | Lower-middle | 57.60 | 3.22 | 103.02 | 158.87 | 1060.14 |
| Viet Nam | Lower-middle | 65.74 | 1.68 | 17.47 | 67.18 | 622.90 |
| *Europe & Central Asia* | | 66.65 | 1.73 | 14.41 | 23.8 | 651.39 |
| Albania | Upper-middle | 68.86 | 1.94 | 7.42 | 17.79 | 519.94 |
| Armenia | Upper-middle | 66.64 | 1.74 | 20.95 | 21.02 | 662.41 |
| Austria | High | 70.6 | 1.50 | 3.20 | 7.66 | 380.67 |
| Azerbaijan | Upper-middle | 63.01 | 1.84 | 21.05 | 48.27 | 994.95 |
| Belarus | Upper-middle | 65.06 | 1.66 | 12.89 | 15.53 | 699.88 |
| Bosnia and Herzegovina | Upper-middle | 66.96 | 1.25 | 3.57 | 12.24 | 637.45 |
| Bulgaria | Upper-middle | 64.58 | 1.56 | 13.25 | 20.36 | 831.04 |
| Croatia | High | 68.21 | 1.34 | 4.55 | 8.31 | 540.02 |
| Cyprus | High | 69.9 | 1.34 | 2.07 | 14.27 | 465.97 |
| Czechia | High | 68.6 | 1.71 | 2.70 | 18.57 | 484.62 |
| Estonia | High | 68.13 | 1.57 | 3.34 | 14.11 | 528.39 |
| Georgia | Upper-middle | 64.49 | 2.01 | 41.47 | 20.49 | 731.02 |
| Greece | High | 69.91 | 1.40 | 5.22 | 20.58 | 423.98 |
| Hungary | High | 66.79 | 1.41 | 11.64 | 9.55 | 615.22 |
| Italy | High | 71.24 | 1.30 | 4.20 | 9.11 | 352.55 |
| Kazakhstan | Upper-middle | 63.16 | 2.45 | 15.53 | 33.35 | 805.99 |
| Kyrgyzstan | Lower-middle | 64.93 | 2.61 | 39.69 | 27.24 | 715.95 |
| Latvia | High | 66.32 | 1.63 | 12.94 | 19.30 | 605.04 |
| Lithuania | High | 66.56 | 1.54 | 6.40 | 17.48 | 581.02 |
| Luxembourg | High | 70.99 | 1.40 | 5.87 | 15.40 | 341.91 |
| Montenegro | Upper-middle | 66.49 | 1.60 | 6.14 | 10.41 | 696.91 |
| North Macedonia | Upper-middle | 65.38 | 1.44 | 6.41 | 12.17 | 876.61 |
| Poland | High | 68.12 | 1.39 | 3.79 | 19.30 | 520.64 |
| Portugal | High | 70.21 | 1.25 | 6.37 | 33.98 | 375.80 |
| Republic of Moldova | Lower-middle | 65.15 | 1.25 | 17.29 | 28.63 | 654.76 |
| Romania | High | 66.36 | 1.59 | 15.15 | 27.18 | 644.92 |
| Russian Federation | Upper-middle | 63.74 | 1.72 | 12.40 | 34.50 | 691.34 |
| Serbia | Upper-middle | 66.33 | 1.43 | 9.28 | 14.79 | 765.56 |
| Slovakia | High | 67.61 | 1.53 | 5.42 | 23.52 | 557.72 |
| Slovenia | High | 70.38 | 1.55 | 6.47 | 14.20 | 392.28 |
| Tajikistan | Low | 61.84 | 3.07 | 21.82 | 69.15 | 1011.92 |
| Turkey | Upper-middle | 67.81 | 1.48 | 28.98 | 23.95 | 496.86 |
| Turkmenistan | Upper-middle | 62.87 | 2.92 | 48.45 | 42.06 | 808.34 |
| Ukraine | Lower-middle | 64.62 | 1.38 | 20.98 | 37.32 | 857.92 |
| Uzbekistan | Lower-middle | 60.88 | 2.44 | 31.90 | 54.96 | 1253.49 |
| *Latin America & Caribbean* | | 65.69 | 1.96 | 74.94 | 54.87 | 551.17 |
| Antigua and Barbuda | High | 66.62 | 1.41 | 41.38 | 49.04 | 583.14 |
| Argentina | Upper-middle | 66.79 | 2.00 | 53.98 | 76.10 | 513.56 |
| Barbados | High | 66.70 | 1.42 | 53.69 | 48.47 | 567.66 |
| Bolivia | Lower-middle | 63.11 | 3.44 | 170.34 | 124.52 | 668.13 |
| Chile | High | 69.23 | 1.65 | 21.40 | 28.73 | 412.45 |
| Costa Rica | Upper-middle | 69.38 | 1.67 | 30.11 | 21.46 | 398.15 |
| Cuba | Upper-middle | 68.43 | 1.46 | 50.51 | 41.16 | 457.98 |
| Dominica | Upper-middle | 63.12 | 1.66 | 104.55 | 55.82 | 713.43 |
| Dominican Republic | Upper-middle | 64.22 | 2.48 | 100.45 | 57.31 | 601.61 |
| Ecuador | Upper-middle | 66.74 | 2.40 | 75.27 | 61.66 | 501.76 |
| El Salvador | Lower-middle | 65.53 | 2.01 | 39.46 | 56.64 | 476.60 |
| Grenada | Upper-middle | 64.03 | 1.81 | 56.12 | 63.24 | 695.11 |
| Guyana | Upper-middle | 58.37 | 2.10 | 169.64 | 101.05 | 870.52 |
| Jamaica | Upper-middle | 66.51 | 1.48 | 76.77 | 33.46 | 543.78 |
| Nicaragua | Lower-middle | 65.78 | 2.28 | 40.46 | 42.83 | 624.58 |
| Panama | High | 69.24 | 2.38 | 70.13 | 43.40 | 367.79 |
| Peru | Upper-middle | 69.90 | 2.42 | 74.31 | 76.09 | 331.45 |
| Suriname | Upper-middle | 62.93 | 2.11 | 116.92 | 65.74 | 600.81 |
| Trinidad and Tobago | High | 65.18 | 1.53 | 47.81 | 35.75 | 577.00 |
| Uruguay | High | 67.45 | 1.90 | 22.66 | 35.14 | 496.24 |
| Venezuela | Upper-middle | 65.44 | 2.19 | 104.25 | 44.51 | 524.09 |
| *Middle East & North Africa* | | 65.69 | 1.96 | 74.94 | 54.87 | 551.17 |
| Algeria | Lower-middle | 66.30 | 2.54 | 72.02 | 31.51 | 631.29 |
| Bahrain | High | 66.59 | 1.35 | 38.39 | 28.21 | 666.01 |
| Djibouti | Lower-middle | 58.94 | 3.71 | 377.79 | 323.37 | 633.69 |
| Egypt | Lower-middle | 62.47 | 2.63 | 35.65 | 49.85 | 913.17 |
| Iran | Upper-middle | 66.83 | 1.92 | 15.88 | 23.79 | 508.93 |
| Iraq | Upper-middle | 63.38 | 2.70 | 36.76 | 25.18 | 709.21 |
| Kuwait | High | 70.25 | 1.37 | 5.12 | 38.20 | 362.47 |
| Lebanon | Upper-middle | 66.04 | 2.59 | 16.28 | 33.29 | 596.48 |
| Libya | Upper-middle | 65.32 | 1.40 | 33.45 | 27.26 | 541.83 |
| Malta | High | 71.07 | 1.47 | 10.97 | 20.57 | 354.06 |
| Morocco | Lower-middle | 63.83 | 2.13 | 93.31 | 52.26 | 722.26 |
| Oman | High | 64.76 | 2.37 | 18.25 | 57.76 | 763.37 |
| Palestine | Lower-middle | 64.42 | 2.99 | 16.08 | 31.68 | 716.43 |
| Qatar | High | 66.27 | 1.70 | 22.80 | 29.77 | 781.99 |
| Saudi Arabia | High | 64.48 | 1.44 | 53.75 | 46.80 | 607.94 |
| Syrian Arab Republic | Low | 63.89 | 2.26 | 21.40 | 31.21 | 726.60 |
| Tunisia | Lower-middle | 67.44 | 1.79 | 32.95 | 24.61 | 529.75 |
| United Arab Emirates | High | 64.38 | 1.14 | 20.78 | 69.25 | 693.71 |
| Yemen | Low | 58.59 | 3.90 | 179.65 | 98.57 | 793.45 |
| *South Asia* | | 63.24 | 2.86 | 192.26 | 101.04 | 678.5 |
| Afghanistan | Low | 54.11 | 5.39 | 269.87 | 140.38 | 1027.22 |
| Bangladesh | Lower-middle | 64.53 | 1.82 | 248.08 | 102.42 | 543.53 |
| Maldives | Upper-middle | 69.11 | 2.32 | 81.33 | 30.21 | 455.66 |
| Nepal | Lower-middle | 61.51 | 2.05 | 277.35 | 136.00 | 721.21 |
| Pakistan | Lower-middle | 57.20 | 3.72 | 241.73 | 191.86 | 814.48 |
| Sri Lanka | Lower-middle | 66.95 | 1.83 | 35.17 | 40.32 | 508.88 |
| *Sub-Saharan Africa* | | 56.65 | 4.15 | 258.65 | 426.11 | 669.17 |
| Angola | Lower-middle | 56.75 | 4.97 | 187.35 | 392.88 | 668.95 |
| Benin | Lower-middle | 56.64 | 5.24 | 261.31 | 410.71 | 627 |
| Botswana | Upper-middle | 54.06 | 2.29 | 105.49 | 466.85 | 793.76 |
| Burkina Faso | Low | 54.16 | 5.56 | 221.53 | 493.27 | 657.51 |
| Burundi | Low | 55.50 | 5.63 | 279.05 | 474.17 | 658.11 |
| Cabo Verde | Lower-middle | 64.62 | 2.30 | 59.91 | 114.72 | 601.14 |
| Cameroon | Lower-middle | 55.43 | 3.81 | 322.77 | 476.01 | 651.28 |
| Central African Republic | Low | 45.71 | 4.62 | 427.59 | 964.02 | 873.67 |
| Chad | Low | 52.85 | 6.87 | 411.26 | 565.21 | 657.42 |
| Comoros | Lower-middle | 60.34 | 3.02 | 153.24 | 266.69 | 601.67 |
| Congo | Lower-middle | 57.02 | 3.47 | 270.04 | 375.14 | 753.46 |
| Côte d'Ivoire | Lower-middle | 56.19 | 4.35 | 254.41 | 457.03 | 615.89 |
| Democratic Republic of the Congo | Low | 56.34 | 4.60 | 344.68 | 391.14 | 681.47 |
| Equatorial Guinea | Upper-middle | 57.21 | 3.49 | 144.94 | 434.63 | 626.03 |
| Eritrea | Low | 55.86 | 3.90 | 434.60 | 464.75 | 729.17 |
| Ethiopia | Low | 60.22 | 4.47 | 207.75 | 302.19 | 553.4 |
| Gabon | Upper-middle | 58.82 | 2.82 | 185.96 | 281.76 | 713.47 |
| Gambia | Low | 58.32 | 4.03 | 404.93 | 378.82 | 674.07 |
| Ghana | Lower-middle | 58.22 | 3.16 | 177.54 | 360.31 | 661.86 |
| Guinea | Low | 53.87 | 4.73 | 466.02 | 501.69 | 706.52 |
| Guinea-Bissau | Low | 53.61 | 4.02 | 242.22 | 498.58 | 817.45 |
| Kenya | Lower-middle | 58.25 | 3.12 | 280.58 | 417.77 | 595.97 |
| Lesotho | Lower-middle | 45.24 | 2.56 | 319.62 | 1022.4 | 978.94 |
| Liberia | Low | 56.89 | 3.49 | 526.21 | 418.28 | 585.11 |
| Madagascar | Low | 57.60 | 3.99 | 272.46 | 357.73 | 722.63 |
| Malawi | Low | 56.58 | 3.65 | 211.08 | 455.74 | 621.05 |
| Mali | Low | 54.26 | 6.04 | 366.22 | 402.54 | 639.63 |
| Mauritania | Lower-middle | 62.17 | 3.59 | 458.46 | 250.95 | 522.73 |
| Mozambique | Low | 50.80 | 4.89 | 184.24 | 716.43 | 705.76 |
| Namibia | Upper-middle | 56.77 | 3.08 | 122.67 | 403.73 | 691.5 |
| Niger | Low | 55.06 | 7.44 | 306.64 | 503.28 | 612.79 |
| Nigeria | Lower-middle | 56.04 | 4.68 | 232.63 | 455.30 | 567.09 |
| Rwanda | Low | 59.83 | 3.53 | 238.10 | 275.78 | 616.97 |
| Sao Tome and Principe | Lower-middle | 62.28 | 2.87 | 126.41 | 168.68 | 736.95 |
| Senegal | Lower-middle | 59.86 | 4.11 | 378.73 | 296.65 | 604.51 |
| Seychelles | High | 64.36 | 2.15 | 55.76 | 107.54 | 633.79 |
| Sierra Leone | Low | 54.06 | 4.07 | 495.29 | 526.25 | 633.68 |
| Somalia | Low | 51.51 | 6.36 | 349.63 | 646.85 | 738.4 |
| South Africa | Upper-middle | 56.18 | 2.17 | 133.25 | 412.49 | 582.35 |
| South Sudan | Low | 54.60 | 5.51 | 184.87 | 480.66 | 563.62 |
| Sudan | Low | 61.42 | 3.66 | 199.96 | 90.78 | 740.38 |
| Togo | Low | 57.01 | 3.78 | 231.95 | 429.06 | 631.57 |
| Uganda | Low | 57.75 | 5.00 | 133.06 | 372.06 | 619.66 |
| United Republic of Tanzania | Lower-middle | 58.73 | 4.67 | 263.75 | 333.62 | 603.51 |
| Zambia | Lower-middle | 55.08 | 4.12 | 130.58 | 463.47 | 740.99 |
| Zimbabwe | Lower-middle | 53.56 | 3.50 | 288.75 | 551.64 | 769.07 |

**Supplementary Table 2.** The Q-statistic and significance level of socioeconomic factors for health status by geographical detector.

| **Health status** | ***Q*-statistic** | ***P*-value** |
| --- | --- | --- |
| *Health-adjusted life expectancy* | | |
| Per capita government health expenditure | 0.527 | <0.001 |
| Universal health coverage | 0.692 | <0.001 |
| Prevalence of undernourishment | 0.319 | <0.001 |
| Basic drinking water | 0.559 | <0.001 |
| Household air pollution | 0.631 | <0.001 |
| Proportion of urban population | 0.579 | <0.001 |
| *Fertility rate* | | |
| Per capita government health expenditure | 0.499 | <0.001 |
| Universal health coverage | 0.702 | <0.001 |
| Prevalence of undernourishment | 0.303 | <0.001 |
| Basic drinking water | 0.587 | <0.001 |
| Household air pollution | 0.629 | <0.001 |
| Proportion of urban population | 0.553 | <0.001 |
| *Maternal mortality ratio* | | |
| Per capita government health expenditure | 0.445 | <0.001 |
| Universal health coverage | 0.697 | <0.001 |
| Prevalence of undernourishment | 0.364 | <0.001 |
| Basic drinking water | 0.507 | <0.001 |
| Household air pollution | 0.696 | <0.001 |
| Proportion of urban population | 0.496 | <0.001 |
| *Communicable disease mortality* | | |
| Per capita government health expenditure | 0.392 | <0.001 |
| Universal health coverage | 0.683 | <0.001 |
| Prevalence of undernourishment | 0.286 | <0.001 |
| Basic drinking water | 0.556 | <0.001 |
| Household air pollution | 0.683 | <0.001 |
| Proportion of urban population | 0.522 | <0.001 |
| *Non-communicable disease mortality* | | |
| Per capita government health expenditure | 0.327 | <0.001 |
| Universal health coverage | 0.349 | <0.001 |
| Prevalence of undernourishment | 0.081 | 0.519 |
| Basic drinking water | 0.244 | <0.001 |
| Household air pollution | 0.198 | 0.048 |
| Proportion of urban population | 0.201 | <0.001 |

**Supplementary Table 3.** Regression coefficients and significance level of socioeconomic factors for health status by spatial lag model.

| **Health status** | **Coefficient** | **Std. Error** | ***Z*-value** | ***P*-value** |
| --- | --- | --- | --- | --- |
| *Health-adjusted life expectancy* | | | | |
| Per capita government health expenditure | 0.881 | 0.333 | 2.644 | 0.008 |
| Universal health coverage | 0.121 | 0.025 | 4.770 | <0.001 |
| Prevalence of undernourishment | -0.068 | 0.024 | -2.840 | 0.005 |
| Basic drinking water | 0.016 | 0.011 | 1.453 | 0.146 |
| Household air pollution | -0.046 | 0.010 | -4.447 | <0.001 |
| Proportion of urban population | 0.009 | 0.013 | 0.689 | 0.491 |
| *Fertility rate* | | | | |
| Per capita government health expenditure | 0.020 | 0.082 | 0.242 | 0.809 |
| Universal health coverage | -0.037 | 0.006 | -5.953 | <0.001 |
| Prevalence of undernourishment | 0.009 | 0.006 | 1.479 | 0.139 |
| Basic drinking water | -0.005 | 0.003 | -1.766 | 0.077 |
| Household air pollution | 0.012 | 0.003 | 4.887 | <0.001 |
| Proportion of urban population | -0.001 | 0.003 | -0.158 | 0.874 |
| *Maternal mortality ratio* | | | | |
| Per capita government health expenditure | -2.650 | 8.167 | -0.324 | 0.746 |
| Universal health coverage | -2.016 | 0.625 | -3.227 | 0.001 |
| Prevalence of undernourishment | 2.091 | 0.590 | 3.545 | <0.001 |
| Basic drinking water | -0.468 | 0.266 | -1.760 | 0.078 |
| Household air pollution | 1.753 | 0.253 | 6.929 | <0.001 |
| Proportion of urban population | 0.592 | 0.319 | 1.859 | 0.063 |
| *Communicable disease mortality* | | | | |
| Per capita government health expenditure | 11.481 | 13.726 | 0.836 | 0.403 |
| Universal health coverage | -2.780 | 1.050 | -2.648 | 0.008 |
| Prevalence of undernourishment | 3.296 | 0.992 | 3.323 | 0.001 |
| Basic drinking water | -1.249 | 0.447 | -2.796 | 0.005 |
| Household air pollution | 2.683 | 0.425 | 6.310 | <0.001 |
| Proportion of urban population | 0.802 | 0.535 | 1.497 | 0.134 |
| *Non-communicable disease mortality* | | | | |
| Per capita government health expenditure | -75.890 | 19.277 | -3.937 | <0.001 |
| Universal health coverage | -1.879 | 1.468 | -1.279 | 0.201 |
| Prevalence of undernourishment | -0.307 | 1.392 | -0.221 | 0.825 |
| Basic drinking water | 0.711 | 0.625 | 1.138 | 0.255 |
| Household air pollution | -0.317 | 0.595 | -0.533 | 0.594 |
| Proportion of urban population | -1.605 | 0.752 | -2.135 | 0.033 |
